# Using the Critical Incident Technique to explore situational emotional labor in sport psychology: methodological proposals

**DOI:** 10.3389/fpsyg.2025.1646782

**Published:** 2025-12-02

**Authors:** Oriane Petiot, Gilles Kermarrec, Jean-François Desbiens, Jérôme Visioli

**Affiliations:** 1 CREAD, UFR Sport and Education Sciences, University of West Brittany, Brest, France; 2 CRIFPE, Faculty of Physical Activity Sciences, Sherbrooke, QC, Canada

**Keywords:** Critical Incident Technique, emotional labor, psychology, sport, physical education, situational approach, mixed method approach

## Abstract

Interventions in sport settings involve intense emotional labor (i.e., effort to adjust emotional responses to professional expectations). This has mainly been explored using quantitative, person-centered approaches that identify general trends. Over the past decades, however, the Critical Incident Technique (CIT) has attracted increasing attention in psychological research as an effective innovative tool for examining emotional labor during crucial circumscribed situations. Following a presentation of the CITs origins and development across different sport fields (e.g., elite sport, physical education), the aim of this article is to put forward CIT-based methodological proposals for analyzing sport interveners’ situational emotional labor. More precisely, we outline two primary data collection modes using questionnaires and interviews. Each approach offers distinct advantages according to the research objectives, context, and participant accessibility. The paper also includes guidelines for both qualitative and quantitative analysis of critical incidents by suggesting how data can be thematically coded and statistically analyzed. In terms of data representation, this article focuses on juxtaposing participant quotes within shared thematic categories and introduces “creative non-fiction” as an innovative narrative technique, presenting data as structured monologues that preserve the authenticity of the participants’ own voices. Finally, the present article outlines future research directions, emphasizing the relevance of the CIT in observational and interventional studies aimed at better understanding and supporting emotional labor among sport interveners. In the case of observational studies, four main future research avenues can be explored by: (a) developing longitudinal observatories to enable long-term tracking of emotional labor; (b) adopting a collective approach to emotional labor by incorporating multiple actors’ points of view during a same critical incident; (c) deepening understanding of the relationships between emotional experience and emotional regulation during critical incidents; and (d) validating a psychometric scale capable of assessing emotional labor during critical incidents across large samples. As for interventional studies, a training program designed to develop adaptive and flexible emotional regulation through CIT use is presented. This program would benefit from being implemented and evaluated, again using the CIT, to reveal its effects on participants.

## 1 Introduction

Sport coaching has been increasingly recognized as an emotion-laden context (Lee et al., 2015) where coaches must regulate multiple emotions, including joy on a win, frustration at a loss, anger at a referee decision, and disappointment with players’ performance (Petiot et al., 2025). In this context, psychological skills have long been acknowledged as key factors in sport performance (e.g., Durand-Bush et al., 2023). These emotional characteristics are also present in Physical Education (PE). Unlike traditional classroom settings, PE takes place in various types of sport facilities, including gymnasiums, stadiums, or swimming pools, and students engage in various physical activities. PE teachers therefore face particular emotional demands in that they have no fixed classroom, must manage dispersed groups of students, and have to adapt constantly to dynamic situations. This environment promotes action and emotional expression rather than silence and immobility, thereby offering multiple opportunities for teachers to experience a variety of emotional states (Petiot et al., 2023a–c). Although sport coaches and PE teachers differ in their objectives, missions, and target audiences, they share common professional features rooted in physical activity settings and deal with unpredictable and high-stakes events. As a result, they are both likely to face intense emotional labor (Lee et al., 2015). These practical and scientific similarities are recognized in sport sciences research and have led scholars to conceptualize emotional labor jointly across both contexts (Petiot and Kermarrec, 2025).

Emotional labor in sport settings has attracted growing attention in sport psychology research over the past fifteen years (Lee et al., 2015) and studies have brought valuable insights into the emotional demands of sport professions, including their impact on performance and well-being (e.g., Lee and Chelladurai, 2016). Most of the existing literature has relied on quantitative, person-centered approaches designed to assess the different ways in which individuals tend to regulate their emotions. Often based on validated self-report questionnaires, these approaches have made it possible to identify the antecedents and consequences of emotional labor across different populations and contexts. However, they provide only a partial picture of the phenomenon, since they abstract emotional labor from authentic and crucial situations. In other words, significant methodological challenges persist regarding the investigation of emotional labor *in situ*, particularly in intense or clearly circumscribed situations (Petiot and Kermarrec, 2025). There is thus a growing need to complement person-centered studies with more situational perspectives that consider emotional labor as inseparable from the situation in which it emerges. The present contribution aims to highlight the relevance of the CIT (Flanagan, 1954) in conducting such situational investigations and propose specific methodological proposals for sport settings.

### 1.1 Definition of emotional labor through a psychological lens

Hochschild (1983) initially defined the concept of emotional labor from a sociological perspective, as the effort deployed to modify the intensity or quality of an emotion within a professional context. According to Mann (2004), workers engaged in “people-oriented” professions are often required to manage their emotions carefully, expressing those considered appropriate while holding back or concealing those that are not. Within certain professions, the consequences can be serious. When a professional fails to display the right emotion such as empathy or unintentionally shows an unsuitable one such as boredom, it can have a significant impact on others’ well-being and the continuity of the professional relationship. Emotional labor in the workplace has therefore emerged as an essential skill, yet can also be a major source of work-related stress and burnout (Lee et al., 2015).

More specifically, this author distinguished between two possible positions in emotional labor. First, an active position wherein professionals intentionally seek to regulate their emotions in the workplace. This active position typically involves two main strategies. “Surface acting” implies simulating the expected emotions, thereby creating “emotional dissonance” (Mann, 2004) between the displayed emotions and those genuinely felt. By way of example, a sport coach might conceal genuine frustration toward players to avoid escalation. Conversely, “deep acting” involves attempting to truly alter the emotional experience. An upset coach, for example, may seek to reframe a situation so as to feel genuine satisfaction instead. Second, professionals may adopt a passive position in which they spontaneously and effortlessly feel the emotions deemed appropriate for the situation. This passive position has led to the identification of a third strategy, known as “genuine expression” (Ashforth and Humphrey, 1993), which represents the automatic and sincere expression of the felt emotions. A coach may genuinely feel and express joy in response to a player’s success, for example.

Nearly twenty years after the initial conceptualization of emotional labor (Hochschild, 1983), a psychological perspective was adopted with the concept of “emotional regulation.” Emotional regulation refers to individuals’ endeavors to influence the emotions they feel and express. This process may be automatic or controlled, conscious or unconscious (Gross, 1998). More precisely, emotional regulation may involve either a decrease (“down-regulation”) or an amplification (“up-regulation”) of emotions. Two main types of strategies have been identified: (1) those targeting the antecedents of the emotional response by acting on pre-response information and (2) those that modify one or more components of the already generated emotional response (expressive, cognitive, physiological) (Gross and John, 2003). In this respect, Grandey (2000) conceptualized emotional labor according to a psychological approach, by developing a model for the “situational cues” influencing it. In other words, the situation acts as a cue from which emotions may result. A salient situation is thus characterized by the interaction between a professional and the expectations of their organization (Grandey, 2000). More precisely, the author’s model includes both the individual differences (such as emotional intelligence) and organizational factors (such as supervisor support) impacting emotional labor. More recently, Grandey and Gabriel (2015) have argued that researchers should consider emotional labor as an umbrella term for a process encompassing professional emotional demands (environmental stimulus), emotion regulation (intrapsychic response), and emotional performance (interpersonal behavior).

### 1.2 Conceptualization of sport interveners’ emotional labor using a situational approach

**Figure 1:**
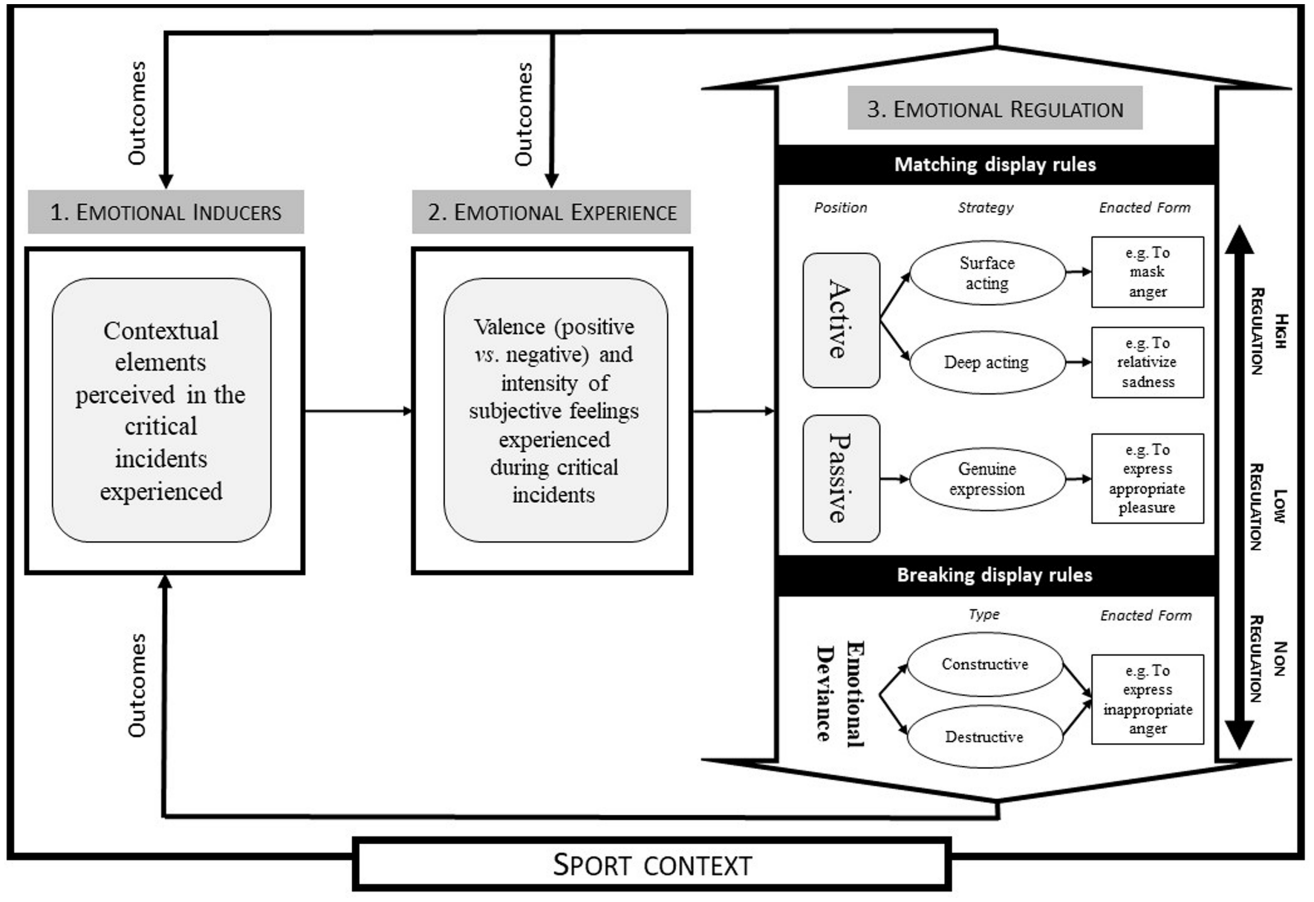
The Situational Emotional Labor and Deviance Model (inspired by Petiot and Kermarrec, 2025).

In line with Grandey (2000), Petiot et al. (2023a,b) developed a situational psychological model of emotional labor anchored in the fields of sport and physical education. It has been conceptualized as the Situational Emotional Labor and Deviance (SELAD) model in a recent theoretical contribution focusing on PE and sport (Figure 1; Petiot and Kermarrec, 2025). More precisely, the model places major importance on the specific context in which emotional labor emerges, as well as on the fact that *in-situ* methods are needed to provide empirical data. As a result, several studies based on this model have involved the collection and analysis of “critical incidents” (Flanagan, 1954) through online questionnaires (e.g., Petiot et al., 2023b) and/or interviews (e.g., Petiot and Kermarrec, 2024a). Using the model developed by Petiot et al. (2023a,b), researchers have identified the “emotional inducers” (i.e., contextual elements perceived in the critical incidents collected) triggering the “emotional experience” and the subsequent enacted forms of “emotional regulation” (involving, for example, amplification or masking). Certain emotional experiences, especially those involving intense negative emotions, may also induce “emotional deviance” (Dahling, 2017) if professionals express sincere but unexpected emotions. In this case, the emotions expressed are congruent with those felt but deviate from the display rules. The SELAD is therefore coherent with the previous approach to emotional regulation, conceptualized by Gross (1998) and Grandey (2000).

In line with this body of work, a critical incident can be defined as a meaningful event for the participant (and potentially those with whom they interact in their professional environment). Similar to the definition proposed by Leclerc et al. (2010), such an event typically occurs in a delicate or challenging situation and is perceived as having the potential to alter the course of events. A critical incident can also be positive for situations in which participants experience a breakthrough after a professional deadlock, for example. More specifically, Leclerc et al. (2010) identified three operational criteria to delineate critical incidents within professional practice: (1) incidents must describe experienced, not imagined, situations; (2) they must be temporally bounded; and (3) they must unfold within an interaction, as a privileged space for the emergence of dilemmas and professional controversies in human-centered professions (Mukamurera et al., 2018).

In addition to these elements, and in line with the SELAD-based conceptualization of emotional labor (Petiot and Kermarrec, 2025), we consider a critical incident to be an emotionally significant situation likely to have major positive and/or negative consequences on an individual’s trajectory. More precisely, a critical incident encompasses three main characteristics. First, it occurs as a consequence of perceived contextual elements, known as “emotional inducers,” which trigger the emotional experience and are often unusual or unexpected. Second, it gives rise to an “emotional experience” of varying intensity and with a positive and/or negative valence. This emotional experience results from a cognitive appraisal of the situation. Third, it leads to a process of psychologically costly “emotional regulation,” reflecting various enacted forms of emotional labor or deviance. Such emotional regulation, in turn, influences both emotional inducers and emotional experience. In the case of emotional deviance, the professional fails to regulate their emotions, resulting in constructive or destructive deviance depending on the outcome (Galperin, 2012).

### 1.3 Methodological challenges in studying situational emotional labor in sport settings

Most studies on emotional labor in sport fields focus on its antecedents and consequences using a psychometric scale and person-centered approach. Antecedents can include positive affectivity (Lee and Chelladurai, 2016), emotional intelligence (Lee and Woo, 2017), and commitment to emotional display rules (Kim et al., 2019). Regarding the consequences, the relationship between emotional labor and burnout has been particularly investigated (Larner et al., 2017; Ha et al., 2021). Several studies have also added the variable(s) of coaches’ turnover intention and/or actual turnover (Larner et al., 2017). Further studies, such as that of Seo et al. (2023), have analyzed other consequences, revealing that coaches’ emotional labor positively predicted their work engagement, but negatively predicted presenteeism. Recently, researchers have adopted a more context-centered approach to emotional labor, using psychometric scales to examine its contextual antecedents. For example, Lee et al. (2017) investigated emotional display expectations among high school teacher-coaches and found role-based differences. Teaching demands more frequent displays of positive emotions (e.g., friendliness), while coaching allows greater expression of negative ones (e.g., frustration, anger). Correspondingly, the teacher-coaches reported a higher use of surface acting in a teaching context and one of genuine emotional expression during coaching. Moreover, Richards et al. (2020) emphasized the role of perceived organizational support, showing that greater support correlates with increased deep acting and genuine expression, while lower support is associated with surface acting, elevated stress, and diminished job satisfaction. These findings highlight the importance of fostering a supportive professional environment that reduces reliance on surface acting and promotes more sustainable emotional regulation strategies.

However, in these studies, researchers investigated the sport interveners’ global perception of emotional labor in the workplace, thereby limiting the possibility of highlighting any complex and various enacted forms in critical situations. In other words, emotional labor was not analyzed in specific and authentic situations, as in a situational approach where it is inseparable from the situation in which it emerges (Petiot and Kermarrec, 2025). Since its earliest conceptual formulations, Grandey (2000) has introduced the importance of emotional events in the understanding of emotional labor. She showed that these events can be “explored through diary studies in which employees describe events and how they responded to them at the time, and then linking these events to overall emotional labor and stress. Observational or laboratory studies may also be used to explore the idea that emotional events influence emotional labor” (p. 103). In sport settings, Carlstedt’s (2004) Theory of Critical Moments posited that mental factors are most crucial during specifically delineated psychologically significant competition periods and contributes to explaining when and why intrusive cognitions influence performance. While initially applied to athletes and competitive environments, this theory can equally shed light on how sport professionals manage emotionally loaded situations, such as injury management, team conflict, failure, or ethically charged decisions under pressure. Although such moments are central to the lived experiences of sport professionals, they are often overlooked in research due to methodological constraints.

The CIT offers a promising approach (Flanagan, 1954) to address this gap. By focusing on emotionally salient events, it helps uncover professionals’ subjective experiences in action. Drawing on previous empirical contributions, the aim of the article is to present CIT-based methodological proposals for investigating the situational nature of sport interveners’ emotional labor, which remains under-theorized and under-measured in sport psychology (Petiot and Kermarrec, 2025). For this purpose, we have first shown the origins and subsequent variations of CIT (Flanagan, 1954) before, finally, formulating directions for future CIT research on emotional labor.

## 2 Presentation of the Critical Incident Technique

The term *incident*, from the Latin *incidere* meaning “to fall into” or “to happen,” refers to an event that interrupts the normal course of action and calls for a decisive response. Combined with *critical*, implying potential disruption or risk, it echoes Flanagan’s (1954) original notion of the “critical incident,” later refined through decades of methodological development (Sharoff, 2008). In this respect, the aim of the present article is to add to this body of work by proposing a redefinition of the CIT, through an analysis of professionals’ emotional labor that extends contributions centered on the subjective nature of the incident, the perceived factors surrounding it, and the criticality of events leading up to it. Developed through the work of Chell (1998), this approach suggests that the CIT can facilitate the investigation of significant occurrences, which may be events, incidents, processes, or issues identified by professionals. This interpretive version of the CIT enables researchers to explore the nature of the incident and why it is considered significant, as well as how it has been managed and its perceived consequences. The objective is to gain an understanding of the incident from the individual’s point of view, taking into account their beliefs, feelings, and actions (Gremler, 2015). We have illustrated our approach within the field of sport psychology.

### 2.1 Origins of the Critical Incident Technique

The CIT was developed by psychologist John Flanagan during World War II as part of the U.S. Air Force’s aviation psychology program. Designed to identify factors underlying success and failure in pilot training, it consists of systematic procedures for collecting direct behavioral observations to address practical problems and derive psychological principles (Gremler, 2015). By asking trainees and supervisors to report significant events, Flanagan identified key behaviors distinguishing effective from ineffective performance.

This method gained considerable popularity after the War, particularly with the setting-up of the American Institute for Research, which incorporated the principles of the CIT into the qualitative analysis of human behavior. Flanagan subsequently formalized the technique through publications outlining its principles and key stages. According to him, “an incident is considered critical only if, in the observer’s opinion, it relates to an important aspect of the work and includes behavior which is outstandingly effective or ineffective with respect to the specific situation” (Flanagan, 1949, p. 42). Ultimately, an incident can be any observable human activity that is self-sufficiently complete, thereby allowing inferences and predictions to be made about the person performing the act (Flanagan, 1954).

More specifically, according to Flanagan (1954), the CIT is based on five major steps: (a) determining the general aim; (b) specifying plans and instructions; (c) collecting data; (d) analyzing data; and (e) interpreting results. The analysis therefore encompasses cognitive, affective, and behavioral dimensions, addressing both the content of what is learned and the process through which learning occurs (Serrat, 2017).

### 2.2 Development of the Critical Incident Technique in various fields

Since the 1970s, the CIT has undergone multiple adaptations across a variety of professional domains. An early literature review (Fivars, 1973) already identified more than 600 studies on use of the technique in the military, nursing, management, and education sectors. Over the following decades, more specific frameworks emerged, aiming to tailor the CIT approach to the professional realities of each discipline. For instance, Woolsey (1986) proposed a contextualized version for counseling, while Duffy (1983) redefined the CIT as a training tool for decision-making in business. An analysis of customer dissatisfaction in public transportation (Edvardsson, 1998) further illustrates the diversification of its applications. The 21st century has seen a resurgence of interest in the technique, as evidenced by several literature reviews (Serrat, 2017). The CIT is now used to explore effective or ineffective behaviors, to identify facilitating or constraining work factors, and to describe the dynamics of interactions in critical situations. Various contemporary adaptations have been conceptualized.

This is the case for the theory of cultural shocks, developed by Margalit Cohen-Emerique through work with migrant families. The theory draws on lived critical incidents to reveal sensitive zones in intercultural encounters, such as perceptions of the body, time, family structures, or belief systems. Cultural shock thereby becomes a lever for raising awareness of one’s own cultural identity. Often used in training contexts, this technique follows a structured protocol that combines storytelling, collective analysis, and feedback. It highlights the fact that critical incidents are inseparable from the context in which they occur and can reveal sources of conflict such as stereotypes or gender roles (Ramos-Vidal, 2011).

A second notable evolution is the Critical Decision Method (CDM), developed from Klein’s work (Klein and Armstrong, 2004) on decision-making in real-world settings. Grounded in the Naturalistic Decision-Making approach, this method emphasizes the role of intuition and experience in uncertain, complex, and time-pressured environments like sport (Bossard et al., 2022). CDM follows a five-step procedure that builds on the CIT logic to closely examine key decision points. It has been applied in a wide range of sectors to understand how experts make real-time decisions, often without relying on formal data (Trönnberg and Hemlin, 2014). These developments demonstrate that the CIT is far from static but rather continues to evolve in response to contemporary challenges, professional contexts, and emerging research topics.

### 2.3 Critical Incident Technique in sport sciences: an overview

Interest in the CIT has gradually increased in the field of sport sciences. From a theoretical standpoint, Louder and Stevens (2012) demonstrated how critical incidents can disrupt communication, action, and support structures within a sport organization. Their analysis of the motorsport sector highlighted the complexity of interpersonal relationships in such contexts, as well as the intense emotional pressure experienced by individuals, particularly those required to intervene during a crisis. In a further example, Sveinson et al. (2021) emphasized the value of qualitative approaches, such as the CIT, in understanding power relations in sport management and advocated the broader integration of such methods in sport management research.

Beyond these theoretical considerations, the CIT has also been used in interventional studies assessing the impact of programs implemented in sport fields. For instance, in a qualitative evaluation of the *Let us Move It* program, Kostamo et al. (2019) showed how the CIT helped identify the intervention elements perceived by participants as triggers for change. Analysis of post-program interviews revealed the CIT’s value in capturing the moments deemed significant by the young in both the intervention and control group. In addition, Kettunen et al. (2024) employed the CIT to explore critical incidents related to the use of digital coaching technologies. Their findings revealed that such experiences, whether positive or negative, played a central role in users’ motivation to sustain physical activity. This particularly highlighted the importance of perceived support, especially among older populations.

Adopting an observational approach, Greenwell et al. (2007) demonstrated CIT utility in collecting and categorizing customer feedback during sporting events. Their study identified service-related dimensions influencing spectator satisfaction or dissatisfaction and showed that critical expectations varied across sport disciplines and sociodemographic profiles. Furthermore, Dasinger and Solmon (2021) used the technique to explore sources of anxiety in physical activity settings. Their work revealed several emotional triggers, particularly fear of social judgment and feelings of incompetence, likely to impact future participation. By focusing on novice physical education (PE) teachers, Vandercleyen et al. (2014) showed that “organizational critical incidents” were experienced differently by teachers, according to two main factors: their perceived self-efficacy in classroom management and their ability to seize emerging opportunities.

These different studies converge, however, on a shared conclusion: the Critical Incident Technique has proven effective in highlighting emotionally charged and meaningful situations that shape sport actors’ experiences. Recent studies anchored in sport psychology have moreover directly linked the technique to the analysis of emotional labor among athletes (e.g., Petiot et al., 2024) or sport interveners (e.g., Petiot et al., 2025).

### 2.4 Research on sport interveners’ emotional labor using the Critical Incident Technique

Several CIT-based studies have aimed to investigate emotional labor using a situational approach.

First, Petiot et al. (2023a) emphasized the fact that elite coaches experienced frequent and intense emotions, particularly in competitive contexts requiring significant emotional regulation. Through a longitudinal three-season partnership with a professional handball coach, the authors demonstrated that emotional labor is not a peripheral concern but rather a structuring element of daily professional activity. The authors used various *in-situ* methods like the CIT to characterize the coach’s emotional flexibility. Expanding on this research, Petiot et al. (2025) also adopted a situational approach to analyze the emotional labor of ten elite handball coaches during fifteen high-stakes matches. The authors identified 121 crucial moments of emotional labor, revealing four enacted forms: (a) “Sincerely expressing emotions”; (b) “Masking emotions”; (c) “Relativizing emotions”; and (d) “Amplifying emotions.” These forms of enactment were closely related to the emotional experience (positive vs. negative), resulting from emotional inducers, such as “Inappropriate behavior from the players,” “Disagreement with a referee’s decision,” and “Players’ success.”

Second, the CIT has been used to analyze PE teachers’ emotional labor in challenging educational contexts. For example, Petiot et al. (2023b) analyzed 203 critical incidents reported by 61 PE teachers and found that emotional labor is a core component of their teaching activity. Although negative emotional inducers (e.g., student violence) were predominant, the findings also highlighted the importance of positive emotional inducers (e.g., quality of human relationships) in enacted forms of emotional regulation. The technique was also central in studies focusing on specific emotional labor strategies. Petiot and Kermarrec’s (2024a) analysis of PE teachers’ critical incidents revealed four forms of surface acting: masking negative emotions, masking positive emotions, amplifying negative emotions, and amplifying positive emotions. The teachers’ emotional regulation during the critical incidents reflects the nuanced and complex adjustments teachers must make to maintain authority, pedagogical relationships, and classroom climate. However, when emotional regulation fails, emotional deviance may emerge. Petiot and Kermarrec (2024b) documented six cases of emotional deviance through vignettes based on critical incidents occurring in PE teaching. These included acts of revenge, verbal aggression, violent exclusion of students, withdrawal, and breaches of professional rules. The study showed that such deviance typically arises as a last resort, in response to intense and repeated negative emotional experience. In sum, whether in elite sport or PE teaching, the CIT appears as an innovative methodological approach to apprehend emotional labor, as a complement to the numerous quantitative studies based on psychometric scales (Lee et al., 2015).

## 3 Methodological proposals

Our methodological proposals align with the epistemological stance adopted by Petiot and Kermarrec (2025) in conceptualizing the SELAD, which is grounded in critical realism. This paradigm combines critical realism ontology (i.e., the assumption that phenomena exist independently of our perceptions) with constructivist epistemology (i.e., the view that scientific knowledge is actively constructed through interpretation and context) which considers knowledge to be situated, interpretive, and historically embedded (Ryba et al., 2022). Thus, while reality exists independently, our understanding is shaped by conceptual frameworks and sociocultural contexts. This approach differs from positivism and relativist constructivism by rejecting both universal, decontextualized measures and radical, epistemological relativism. Emotional labor is therefore seen as a socially and materially situated phenomenon, emerging from structured interactions within specific contexts. Consistent with this epistemological foundation, the CIT makes a detailed reconstruction of emotional inducers, experience and regulation possible by enabling the integration of complementary methods. In this way, it captures both the subjective meanings and the objective structures that shape professional emotional labor (e.g., Petiot et al., 2025).

### 3.1 Proposals for data collection

#### 3.1.1 Critical Incident Technique using questionnaires

Several studies focusing on emotional labor in PE teaching have been based on questionnaires designed to elicit critical incidents, with the aim of analyzing the emotional inducers experienced in various settings (e.g., Petiot et al., 2015). The initial version of such a questionnaire consisted of three main components: (a) an introductory note outlining the objectives and procedures of the study and guaranteeing participant anonymity; (b) a sociodemographic section with information such as age, gender, years of teaching experience, subject taught, geographical region, and teaching context (e.g., compensatory education); and (c) an open-ended part inviting teachers to report one or more positive or negative critical incidents experienced during their careers, along with the emotions that accompanied these events. Subsequent research has adapted this instrument to suit specific educational contexts. For instance, in a study conducted in a compensatory education environment (Visioli and Petiot, 2023), teachers were prompted in their accounts as follows: “*Please describe, in as much detail as possible, significant events that occurred in the course of your professional activity. These moments may have triggered either positive or negative emotions. The only criterion is that they were meaningful to you and involved students in a compensatory education context*” (p. 6). This open mode of questioning makes it possible to minimize response bias and allows participants to report critical incidents they perceived as emotionally significant, thereby facilitating an initial exploration of situational emotional inducers.

Further research proposed an enriched version of this initial questionnaire so as to analyze other components of the situational emotional labor model (Petiot and Kermarrec, 2025). As noted by Paillé and Mucchielli (2012), qualitative research is marked by a degree of unpredictability, owing to the personal nature of data collection and the use of methods which rely on participants’ verbal accounts. Such uncertainties are an inherent feature of the research process. While the original version of the critical incident questionnaire offered the advantage of non-directiveness, its structure reduced the possibility of capturing the full scope of emotional labor as conceptualized in the model, namely including emotional inducers, emotional experience, and emotion regulation. As a result, the potential for in-depth data analysis remained restricted, particularly beyond the identification of emotional triggers. With a view to addressing these limitations, the researchers further enriched the questionnaire to align it more explicitly with the model while preserving a qualitative orientation (Petiot and Kermarrec, 2024a,b). This revised version invites participants to: (a) describe the context of the event and identify the elements perceived in the situation (emotional inducers); (b) report the emotions experienced during the event (emotional experience), combining open-ended narrative responses with a bipolar scale ranging from −5 (very negative emotions) to +5 (very positive emotions); and (c) explain the strategies used to regulate these emotions during the event (emotion regulation). The sequence of questions follows both the model and the natural structure of participants’ narratives. In practice, respondents tended to begin with contextual descriptions of the event before addressing its emotional consequences. This structure therefore facilitates the collection of richer, more structured data, enabling a more comprehensive analysis of emotional labor within professional sport contexts.

#### 3.1.2 Critical Incident Technique using interviews

To deepen the understanding of situational emotional labor, it is possible to collect sport interveners’ critical incidents by means of interviews. For instance, the authors associated an online questionnaire with interviews in the context of PE teaching (Petiot and Kermarrec, 2024a,b). More specifically, they conducted interviews by screen sharing the five critical incidents reported via the online questionnaire over the course of a school year. The interviews involved discussing each critical incident, in chronological order, for approximately ten minutes. More precisely, the critical incident, as written by the participant, was first read aloud. The teacher was then invited to mentally return to the situation and describe it as though reliving the experience (Table 1).

**Table 1:** Synchronization of the questionnaire responses and interview verbalizations.

Written critical incident	Interview
1. During the first week of school, I gave my first lesson to one of my 7th-grade classes. Although I had been warned, I discovered that the behavior of most of the students was insufferable: arguments, refusing to participate, repeated and inappropriate disruptions… 2. -3 3. I quickly felt annoyed and angry, seeing how difficult they were, even before we’d introduced ourselves, before we had the chance to get to know each other. 4. Internally, I tried to put things into perspective and told myself that they were also like this in other subjects. I tried not to show it and punished some of the students who crossed the line.	Researcher: So, if we go back to the beginning of the school year, could you picture yourself in the situation you describe here [reads the response to the first question]? Do you remember what happened? Teacher: Yes, I remember. They were completely unmanageable. They always had something to say about everything, never agreed on anything. The activity I had suggested did not suit them. They quickly ruined the lesson. The inappropriate behavior of the students, the fact that it kept being repeated during the class… It was very difficult. I’ve had other challenging classes before, but usually you can sense the ups and downs. With them, I could not find any button to press to even slightly spike their interest (…). Researcher: So, how did you feel? You rated your emotional state as −3 in the previous question. Teacher: I felt really irritated. Yes, that’s right. We did not even know each other. I found it really hard. I did not feel hopeless, but I had a feeling the year was going to be tough, and that was before we’d actually started, before I even had a chance to try (…) Researcher: How did you deal with these emotions? [reads the response to the fourth question] Teacher: That’s exactly what I wrote, I remember it very well. I tried to put things into perspective because I did not want their behavior to affect me. At the same time, I hid what I was feeling. I tried to talk to them without raising my voice or getting angry, to show them I wasn’t going to give in.

More generally, for each critical incident, questions were guided according to the three components of the model: emotional antecedents (what was perceived in the situation), emotional experience (what was felt), and emotion regulation strategies (how emotions were managed). These interviews provided a deeper understanding of the emotional labor involved, by adding oral elaboration to the previously collected written responses (Table 2).

**Table 2:** Interview guide (Petiot and Kermarrec, 2024a,b).

Component of emotional labor (Petiot and Kermarrec, 2025)	Questions for teachers
Emotional inducers	Please describe the context of this moment. What happened during it? What were the contextual elements that triggered your emotions?
Emotional experience	How did you feel in this situation? What specific emotions did you feel? How can you describe the emotions you felt?
Emotional labor	How did you regulate your emotions toward the students? What decisions did you make in the course of the action? What were your concerns when you made this decision?

Other studies have sought to analyze emotional labor over longer time frames than those addressed through the critical incidents captured by questionnaires or interviews focusing on single lessons or matches. Their objective was to understand how emotional labor unfolds during critical incidents occurring across an entire professional trajectory (e.g., Petiot et al., 2024). These career-long studies involved a timeline-based and self-confrontation method (Visioli et al., 2025), inspired by “life course” interviews previously developed within the enactive framework of the course-of-action research program (e.g., Hauw, 2013). More specifically, each interview began with the projection of a timeline representing the participant’s career. The interview then returned to the starting point of the career, with participants invited to present the significant moments experienced in terms of: (a) what they perceived (emotional inducers); (b) what they felt (emotional experience); and (c) how they regulated the emotions (emotion regulation). Ultimately, the career-long, timeline-based and self-confrontation interview method offers a valuable way to investigate emotional labor in sports contexts over extended periods of time. In studies using this approach, the data collected allow an exploration of the situational emotional labor dynamics occurring in the critical incidents experienced.

### 3.2 Proposals for data analysis and representation

#### 3.2.1 Qualitative data analysis of critical incidents

Over the past decade, a qualitative approach has developed and refined a CIT-based methodological approach to examining emotional labor in educational and sport settings. Early studies (e.g., Petiot et al., 2015) focused on inductively identifying the emotional antecedents perceived by participants, without initially targeting specific emotional labor strategies. More recent contributions (e.g., Petiot et al., 2025) have expanded the analytical framework to include the three components of the situational model (emotional inducers, emotional experience and emotional regulation). The analysis conducted by the authors followed a process drawing on the principles of grounded theory (Charmaz and Thornberg, 2020). This approach aims to generate theoretical categories from empirical data, rather than testing pre-established hypotheses, thereby aligning with an inductive and exploratory research perspective.

First, all incidents were classified based on the emotional valence reported by participants: incidents associated with positive emotional experiences were separated from those linked to negative ones. Following this initial classification, each subset of incidents underwent a first round of categorization for the components investigated. Regarding emotional regulation, previous studies applied a mixed deductive-inductive approach, in which the first step involved deductively identifying the emotional labor strategies enacted during the critical incidents collected. Drawing on Hochschild’s (1983) contributions, the researchers identified action verbs used by PE teachers to describe how they managed each emotional situation (Table 3).

**Table 3:** Typical action verbs associated with emotional labor strategies.

Position	Active		Passive
Strategy	Surface acting	Deep acting	Genuine expression
Action verbs	Pretending Hiding emotions Exaggerating Filtering Lying	Keeping it together Keeping calm Changing emotions Struggling against felt emotions	Showing emotions Communicating feelings Being transparent Expressing feelings

The second step consisted in inductively identifying enacted forms of emotional regulation, as in the contribution of Petiot and Kermarrec (2024a) concerning the strategy of surface acting: masking positive emotions, masking negative emotions, amplifying positive emotions, and amplifying negative emotions. This second step involved a subcategorization process. In other words, incidents were reread carefully to inductively identify recurring features that could refine previous thematic categories.

Finally, the reliability of the categorization system was assessed through a dual validation process. First, intra-rater reliability was tested: a sample of critical incidents was re-coded by the same researcher several days after the initial coding to examine coding stability over time. Second, inter-rater reliability was assessed by having an independent researcher – unfamiliar with the initial categorization – code the same sample using the developed coding grid. Agreement rates between the two coders were calculated to evaluate the robustness of the system. At this stage, two complementary approaches can be highlighted: one based on percentage agreement, which provides a general indication of consistency; the other relying on Cohen’s Kappa coefficient, which accounts for agreement occurring by chance and offers a more statistically robust assessment of inter-rater reliability.

#### 3.2.2 Quantitative data analysis of critical incidents

Data quantification is also possible when emotional labor experiences are collected through CIT. Following the inductive categorization of the model components (e.g., emotional inducers), a systematic process of quantification can be conducted. Petiot et al. (2023c) have highlighted three main benefits of quantifying qualitative data to analyze emotional labor using the CIT, corresponding to three procedures: (a) weighting categories to establish their relative importance; (b) comparison of category prevalence across different contexts; and (c) statistical evaluation of relationships between the model components.

The first procedure involves calculating the absolute and relative frequencies of each identified category and subcategory. This makes it possible to prioritize the thematic elements emerging from the dataset by determining their relative weight in the overall corpus. For example, in a study on emotional inducers among teachers in compensatory education, Visioli and Petiot (2023) quantified the number of occurrences associated with each emotional inducer category to highlight the most salient sources of emotional experience. This descriptive statistical approach allowed the researchers to identify the category most frequently mentioned by the participants. The study revealed 130 negative occurrences grouped into six categories (e.g., student violence towards the teacher).

The second procedure involves comparing category frequencies across different contexts. This cross-contextual comparison provides insight into how specific emotional labor strategies and emotional experiences vary depending on environmental constraints. For instance, Petiot et al. (2023c) compared the emotional regulation strategies employed by PE teachers working in compensatory education with those used by PE teachers working with students with disabilities. By calculating and comparing the absolute and relative frequencies of each strategy in both contexts, the authors identified both convergences and divergences. While the category “Managing negative emotions generated by unpleasant situations” was the most frequently reported in both contexts, certain passive enacted forms of emotional regulation were more prevalent among teachers working with students with disabilities.

The third procedure aims to identify statistical relationships between different components of the model. To do so, it is possible to create a contingency table mapping the co-occurrence of specific categories across the incidents described. For example, Petiot et al. (2025) analyzed the relationships between emotional inducers and emotional regulation among elite handball coaches during 121 moments of emotional labor occurring in crucial matches. They used the R package *GDAtools* (Robette, 2022) to perform chi-square tests, based on observed and expected frequencies, and visualized the results using mosaic plots. In addition to the chi-square statistic, they calculated Cramér’s V to assess the strength of association between variables, and Pearson’s phi coefficient to assess the relationship between specific modalities. The results of the Chi-square test demonstrated that the relationship between the enacted forms of emotional regulation and the emotional inducers was significant, with a strong effect size. The coaches’ emotional regulation was, therefore, dependent upon the emotional inducers perceived during the matches. When the coaches’ emotions were induced by negative player behavior, they were likely to mask their emotions (*phi* = 0.41) or to relativize them (*phi* = 0.28). However, when their emotions were induced by refereeing decisions, they primarily amplified them (*phi* = 0.45). Finally, when the coaches’ emotions were induced by their players’ success, they tended to express them sincerely (*phi* = 0.36).

#### 3.2.3 Toward a deep representation of critical incidents

In most previous research on emotional labor results are presented by juxtaposing the verbatim extracts of different participants according to a shared thematic category, following the initial approach of Petiot et al. (2015, 2014). This representation mode highlights similarities and contrasts in how individuals experience and describe emotional labor, emphasizing either the recurrence of specific emotional patterns or the diversity of teachers’ interpretations and regulatory efforts within comparable professional contexts. Such data representation is usually used in qualitative research, including other studies on emotions in sport fields (e.g., Descoeudres et al., 2022).

A second more innovative and deep representation mode involves using *creative non-fiction* (Cavallerio, 2022). This has been used in research on emotional labor among sport psychologists (Hings et al., 2018) and PE teachers (Petiot and Kermarrec, 2024a,b). It consists in adopting a narrative-based format by crafting portrait-vignettes that remain as close as possible to the teachers’ own words (Cavallerio, 2022). According to Phoenix et al. (2010), when adopting a narrative approach, researchers may assume the role of either “story analyst” or “storyteller.” In the case of CIT-based research, authors adopted the role of story analysts, meaning that the narratives were treated as data that were subjected to a systematic, rigorous, and scientifically grounded analysis. The vignettes were written in the form of first-person monologues, allowing the reader to access the situation through the teacher’s own voice without simplification or dramatization. In accordance with the situational model of emotional labor (Petiot et al., 2025), each vignette represented a single critical incident and was based on a tripartite structure: (a) the emotional inducers perceived by the teacher, (b) the emotional experience, and (c) the emotional regulation strategy employed (Table 4).

**Table 4:** Example of creative non-fiction related to a critical incident experienced by Miguel (Petiot and Kermarrec, 2024a).

Masking positive emotions: Miguel’s story
*Emotional inducers*
I believe I’ve had one of the most difficult classes I’ve ever had this year. I’ve been teaching in a priority education investment school for 20 years now but feel that it’s one of the first times I’ve had to manage such incidents, conflicts, and inappropriate behaviors. I was also head teacher of the class, so not only did I have to deal with specific PE problems, but also with those that occurred with my colleagues teaching other school subjects. In March, we presented the beginning of our theater show to about fifty middle and high school students. We were not at all certain that the class would engage given the difficulties experienced since the start of the year. Fortunately, they did. The students were focused and conformed to the rules. Moreover, they were enthusiastically applauded for their performance, and many students came out at the end to congratulate them. These life-battered students with their chaotic journeys experience few moments during which they have the opportunity to feel proud.
*Emotional experience*
I was really happy to witness such a successful moment for them. The beginning of the year had been so difficult that sharing a positive moment with them was a great relief. I was proud of them. I was proud of us as teachers, too. After several months, we managed to help them feel comfortable in their role as a student. All the teachers really struggled with this class, and we finally had a positive result to reward our efforts. In all the years I’ve spent in a priority education investment school, I’ve had a number of positive experiences, but they often come after a series of difficulties, even major failures. This is also surely what makes them beautiful and probably explains why I feel such positive emotions. When we think back to the number of incidents that have occurred since the beginning of the year, we tell ourselves that a moment like this is salutary. It’s really satisfying. For them. And for us.
*Emotional regulation*
Nonetheless, in such a situation, I need to tread very carefully. Obviously, I wanted to tell them that I was happy with them. I wanted to boost them. And I did, a little. But I could not show or say everything I wanted to. It was so difficult with them… I think you have to feel some satisfaction, but not too much. I had to stay in control; the difficult context, the altercations and the conflicts do not go away. I still have a class that distrusts me. I still have a class that does not believe everything I say. These students are so suspicious of teachers that if you are too positive, it means you are hiding something, that it’s not true. My intention is truly to uplift them. For that, however, you have to be strategic. Yelling does not work, but I’m not sure excessive praise does either. In fact, I’m still working out what I can or cannot say, what I should or should not hide.

### 3.3 Limitations of the Critical Incident Technique and necessary caution

Despite its undeniable contributions to the understanding of emotional labor through authentic and significant situations, the CIT presents several limitations that must be taken into account.

First, while this method allows access to clearly defined, memorable, and richly descriptive events, such incidents may not always align with the overall emotional tone of a professional experience (Simonton et al., 2022). An isolated incident may fail to reflect the broader emotional climate of a work context, thus raising questions about the representativeness of the reported event. What occurs day by day, in the banality of habitual circumstances, operates through accumulation and self-evidence with variation and repetition intertwining, even without conscious awareness. It is within this ordinary flow that ways of seeing the world, ways of thinking, and ways of acting are internalized, learned through immersion and imitation.

Second, the CIT relies on retrospective narrative accounts, which are subject to the risks of memory reconstruction. Although this bias can be mitigated through the use of recall aids, such as timelines, video recordings or written traces (Petiot et al., 2024), the inherent subjectivity of recollection remains a significant methodological limitation. In this respect, the respondent’s motivation and willingness to engage in detailed recollection is also limiting. As noted by Gremler (2015), participants may be reluctant or insufficiently motivated to devote the time and cognitive effort required to fully describe critical incidents. This can result in superficial accounts that limit the depth and richness of the data, potentially undermining the technique’s exploratory potential.

Third, a further limitation lies in the fact that the CIT, as defined in the present contribution, fundamentally depends on the verbalization of experience. As such, it primarily accesses the emotional experience: in other words, what participants can consciously recognize, narrate, and reflect upon. Yet, as highlighted in theoretical debates on emotion, feelings represent only one layer of emotional processes. Many affective dynamics, including bodily states, automatic reactions, or pre-reflective forms of affectivity, often escape conscious awareness and verbal expression. From an enactive perspective on emotion (Colombetti, 2018), emotional episodes are not reducible to mental representations or articulated narratives; they are enacted through the dynamic interplay of brain, body, and environment. Therefore, the CIT may offer limited access to these embodied, pre-conceptual aspects of emotional labor, thereby restricting analysis to the consciously experienced and linguistically mediated dimensions of emotion regulation.

Fourth, the temporal boundaries of a critical incident are sometimes ambiguous in participants’ accounts. A single narrative may refer to a specific moment within a match, an entire lesson, or even a teaching sequence spanning several weeks. According to Myers and Myers (1990), we arbitrarily punctuate the beginning and end of the moments we experience, with no guarantee that others do so in the same way. Inevitably, we each construct our own version of reality, which helps explain why interpretative conflicts arise. This variability complicates comparative analyses and raises important questions concerning the status and unity of the critical incident itself. Researchers are encouraged to address these temporal ambiguities by exploring incidents critically from multiple angles, much like a detective reconstructing an event using witness accounts, so as to clarify contextual elements, temporal boundaries, and the sequence of actions involved.

Finally, there is an inherent difficulty in determining what qualifies as a “critical” incident. What one participant considers critical may not be critical for another, depending on personal sensitivity, professional norms, or contextual expectations. This subjectivity underscores the importance of being highly explicit and consistent in defining inclusion criteria of chosen CIT, in order to ensure analytical rigor and comparability across narratives.

## 4 Future directions for analyzing emotional labor using the Critical Incident Technique

### 4.1 Future directions for observational studies

#### 4.1.1 Developing longitudinal observatories for long-term tracking of emotional labor

Second, the development of longitudinal observatories would enhance our capacity to track the evolution of emotional labor over time. Employing the CIT in this context would allow researchers to document sequences of critical incidents and their emotional repercussions, providing insights into how regulation strategies evolve and how they are shaped by contextual and individual factors. While some longitudinal studies have already been conducted, such as year-long investigations in PE (Petiot and Kermarrec, 2024a,b) or career-spanning analyses of critical incidents in elite sport (Petiot et al., 2024), these studies remain scarce and recent. Future research could deepen this line of inquiry by exploring: (a) the real-time dynamics of critical incidents as they unfold over multiple years with the same participants, and (b) retrospective reconstructions of lived incidents throughout an individual’s career trajectory (Hauw, 2013; Visioli et al., 2025).

#### 4.1.2 Adopting a collective approach to examine various actors’ points of view

Third, future research should adopt a collective approach to emotional labor by integrating the perspectives of multiple actors involved in the same critical incident, in line with previous studies on other psychological concepts such as emotional regulation (Campo, 2019), coping (e.g., Le Prince et al., 2018), and self-regulation (e.g., Clayton Bernard and Kermarrec, 2022). Comparing and confronting accounts from different stakeholders, such as coaches, athletes, teachers, or students, could shed light on the intersubjective dimensions of emotional labor and reveal how emotion regulation is negotiated and coordinated within social interactions. Despite its original emphasis on collective viewpoints (Flanagan, 1954), existing CIT-based studies on emotional labor have not yet confronted the perspectives of different participants regarding the same critical incident. This represents a significant gap. Addressing this issue could lead to important findings about how actors align, conflict, or coordinate their emotion regulation efforts in shared situations.

#### 4.1.3 Deepening the relationships between emotional experience and regulation

Finally, it is essential to deepen our understanding of the relationship between emotional experience and emotional regulation within critical incidents. To date, research has mainly examined how emotional inducers relate to the choice of regulatory strategy, but little is known about how the emotional experience itself interacts with emotional regulation. For instance, Scott et al. (2020) observed that much of the emotional labor literature tends to contrast surface and deep acting strategies. Their study with 218 employees revealed that the valence of expressed emotions plays a central role in determining the adaptive or maladaptive effects of emotion regulation. However, in previous sport psychology research, emotional experience is rarely integrated as a variable in emotional labor analysis. It typically serves as a bridge between emotional inducers and emotional regulation, and is often reduced to the positive or negative valence of the recounted incident (e.g., Petiot et al., 2023a). Therefore, there is significant potential to refine the characterization of emotional experiences related to critical incidents, taking into account their valence and intensity. Doing so would offer a more nuanced understanding of emotional labor processes that unfold in sport settings.

#### 4.1.4 Validating a psychometric scale to assess emotional labor within the critical incidents

First, there is a need to develop and validate a robust psychometric instrument capable of assessing emotional labor in response to critical incidents across large samples. Such a tool would allow researchers to quantify emotion regulation patterns and identify the specific strategies that interveners mobilize during these incidents. To ensure theoretical consistency, the development of this questionnaire would benefit from adopting the situational model of emotional labor developed by Petiot et al. (2023a,b) as an underlying framework. This model highlights the different regulatory alternatives available to sport interveners according to situational demands. Further, previous qualitative-dominant studies have shown that each emotional labor strategy may be enacted in various forms. For example, Petiot and Kermarrec (2024a) identified four distinct enacted forms of surface acting among PE teachers, offering a valuable foundation for generating diverse items for this dimension. The situational emotional labor questionnaire used could be constructed and validated in line with similar principles to those of existing sport-specific situational questionnaires (e.g., Kermarrec and Michot, 2007; Roure et al., 2016).

### 4.2 Future directions for interventional studies

#### 4.2.1 A Critical Incident Technique-based program

**Figure 2:**
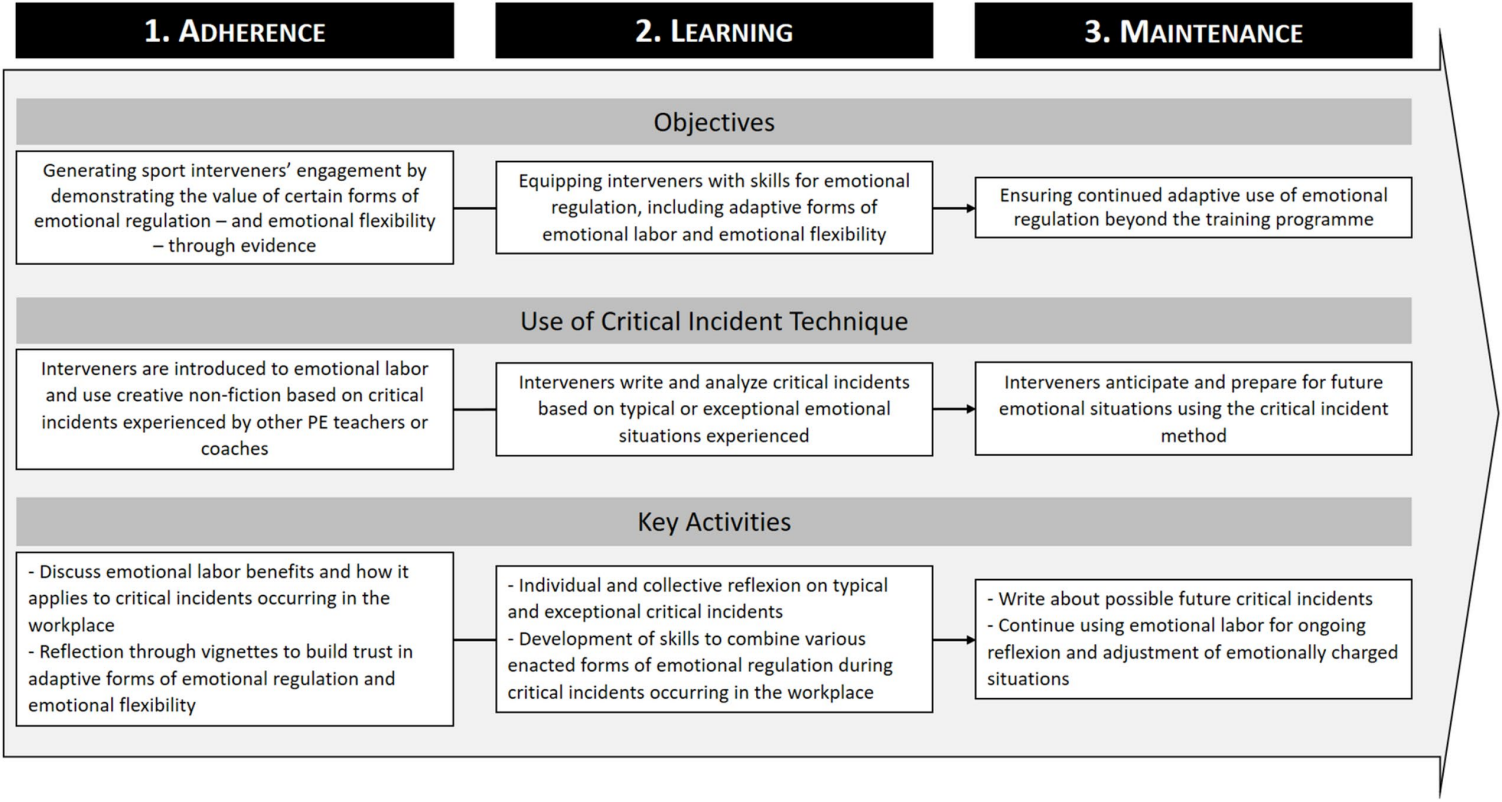
Design of a CIT-based training program for sport interveners’ emotional labor.

The CIT has been recognized as a valuable tool for supporting professional development in various fields (e.g., Joshi, 2018). By encouraging participants to reflect on real-life situations, the method fosters deeper analysis of practice and professional identity. Empirical studies have demonstrated its effectiveness in promoting reflective thinking (Bruster and Peterson, 2013), particularly when combined with structured discussion and writing formats such as journals or blogs. Moreover, Leclerc et al. (2010) formalized principles for using the CIT within a transformative training framework, emphasizing the importance of grounding reflection in lived experience, collegial exchange, and theoretical insight. Their four-step spiral process (individual preparation, group discussion, individual incubation, and collective reflection) offers a structured model for embedding the CIT into professional training programs. More precisely, we could create and implement a training program on emotional labor among sport interveners based on Leclerc et al.’s (2010) work and on findings obtained through use of the CIT in sport psychology. This evidence-based program (Bernier et al., 2025) could be structured according to three key modules: adherence, learning, and maintenance (Figure 2).

The first module would aim to generate engagement and demonstrate the value of emotional labor in managing critical incidents. Interveners could be introduced to the concept of emotional labor and the benefits of certain strategies (genuine expression, deep acting) during emotionally demanding situations (e.g., Lee, 2017). The goal is to help PE teachers and coaches to be aware of the omnipresence of emotional labor in their professional settings (Petiot et al., 2025). To support this module, it would be possible to use the creative non-fiction approach presented in previous studies to facilitate the sharing of experiences. This approach allows interveners to gain insight into how emotional regulation can emerge in practice, as well as how it can positively influence emotional responses. The focus here would be on building interveners’ trust in emotional labor for effectively managing critical incidents in the workplace.

In the second module, interveners would be trained in using the Critical Incident Technique to reflect on specific instances where emotional regulation is crucial. They would be encouraged to describe in writing and analyze critical incidents that represent typical or exceptional emotional experiences in their practice. This process would help them identify when specific forms of emotional regulation are most beneficial and how they can be combined to effectively manage emotionally charged situations. The use of vignettes from the study would serve as a learning resource, enabling interveners to gain perspective and develop strategies based on real-life examples. This module would alternate between individual and collective learning, as recommended by Leclerc et al. (2010). It would focus on equipping sport interveners with practical tools for improving their “emotional flexibility” (Petiot et al., 2025) and response to critical incidents.

The final module would be designed to ensure the continued application of emotional regulation in an adaptive manner beyond the training program. Interveners would be encouraged to anticipate future critical incidents and proactively apply relevant forms of emotional regulation in relation to the specific context of each situation. This module would emphasize the importance of ongoing reflection and adaptability, allowing participants to refine their emotional regulation strategies over time. By continuing to use the Critical Incident Technique, they can remain engaged in self-reflection and adjust their emotional responses as needed, ensuring that emotional flexibility remains an effective tool throughout their careers.

In practical terms, such a program would be delivered over a period of ten to fifteen weeks and combine individual preparation tasks (e.g., reflective writing, vignette analysis) with collective workshops (e.g., discussion groups, feedback sessions). Each module would last approximately two to four one-hour sessions, ensuring a balance between adhesion, learning and maintenance. This modular and iterative structure would make the program adaptable to different professional contexts (e.g., sport coaching, physical education).

To conclude, this evidence-based training program created according to the CIT would provide a structured approach to enhance emotional regulation skills among sport interveners. It would offer a practical framework for addressing emotional labor in sport settings, helping interveners engage with their emotional experiences in a way that improves their professional practice during emotionally charged situations.

#### 4.2.2 A training program evaluated by the Critical Incident Technique

Beyond its use as a pedagogical tool, the CIT also offers valuable potential as a technique for evaluating such a training program in sport psychology. As highlighted by Bernier et al. (2025), the evaluation of psychological interventions remains a critical issue in evidence-based practice. Despite a growing body of literature, meta-analyses have revealed a limited number of intervention studies assessing the effects of psychological programs on performance outcomes (Lochbaum et al., 2022). Such scarcity is even more pronounced when considering elite populations, where precise, high-stakes performance contexts demand rigorous and context-sensitive evaluation tools. In this regard, the CIT offers a promising avenue to document and understand how emotional regulation strategies are mobilized by sport interveners over time and *in situ*.

By collecting and analyzing critical incidents before, during, and after the intervention, it is possible to evaluate how participants make sense of the program, and how their regulation strategies evolve across modules. The CIT allows the identification of changes not only in what interveners do but also in how they adhere to each module – thus offering a richer, experience-based complement to more traditional outcome measures. Furthermore, when applied longitudinally, the CIT can provide insights into the sustainability of changes introduced by the program, thereby addressing the “maintenance” dimension often underexplored in interventional studies.

This qualitative evaluation strategy is particularly well suited to the nuanced and contextual nature of emotional labor in sport settings, where the impact of interventions may not be easily captured by standardized questionnaires alone. For instance, the emergence of adaptive forms of emotional regulation (such as deep acting or genuine expression) can be assessed through the reflective narratives shared by participants, offering evidence of professional growth and increased emotional flexibility. In doing so, the CIT contributes to both formative and summative evaluation, informing continuous program refinement while also documenting its impact.

In sum, integrating the CIT as an evaluative tool not only aligns with calls for more rigorous and ecologically valid assessment methods in sport psychology (Bernier et al., 2025), but also reinforces the program’s experiential foundations. It ensures that evaluation remains grounded in the lived experiences of interveners and responsive to the complex realities of their professional environments.

## 5 Conclusion

Capturing psychological processes in action within sport settings requires methodological approaches that are both situationally grounded and sensitive to subjective experience. The CIT offers a compelling pathway in this regard, enabling researchers to access psychological processes as they unfold within the lived realities of sport professionals. While initially developed to identify determinants of effective performance, its evolution, particularly through interpretive adaptations (Chell, 1998), positions it as a powerful tool for psychological inquiry into complex, dynamic, and context-bound experiences.

In sport psychology, one of the primary contributions of the CIT lies in its ability to situate psychological processes within the specificity of meaningful events. Rather than isolating variables or measuring generalized tendencies, the CIT enables the reconstruction of psychologically significant episodes as narrated by the actors themselves. This narrative anchoring allows researchers to explore how individuals perceive, interpret, and respond to emotionally salient or psychologically taxing moments, whether linked to identity threats, authority challenges, ethical dilemmas, or career transitions.

When applied to emotional labor, the CIT makes it possible to move beyond a person-centered focus on regulation strategies (Lee et al., 2015) by foregrounding the situational conditions in which emotional demands arise (Petiot and Kermarrec, 2025). It brings to light not only the strategies used (e.g., surface acting, deep acting, genuine expression), but also how these strategies are embedded in singular emotional experiences (Scott et al., 2020). The CIT also allows the identification of complex regulation patterns, including the coexistence or succession of multiple strategies within a single incident (Petiot and Kermarrec, 2024a,b), thereby revealing the adaptive, non-linear nature of emotional labor in sport settings.

More broadly, the CIT contributes to sport psychology by offering a methodological bridge between qualitative and quantitative approaches (e.g., Petiot et al., 2025). Through its potential integration in longitudinal designs, or mixed method approaches, it supports the generation of ecologically valid data that retain both depth understanding and generalizability. This versatility is especially relevant in sport contexts, where psychological phenomena are embedded in performance cultures, institutional constraints, and social expectations that are difficult to capture through standardized tools alone.

Beyond emotional labor, the CIT provides a means to access a range of underexplored psychological constructs, such as moral dilemmas, identity ruptures, relational tensions, or existential questioning, that often emerge in the form of “critical moments” (Carlstedt, 2004) and to resist linear or uniform explanations. By engaging directly with the meaning-making activity of participants, the CIT aligns with constructivist epistemologies that view psychological experience as emerging from person-environment coupling.

The CIT therefore constitutes a valuable and underutilized methodological option in sport psychology. Its capacity to foreground situational complexity and subjective meaning makes it particularly suited for investigating the nuanced, context-sensitive nature of psychological phenomena in applied settings. As such, we advocate a broader adoption of the CIT, not only to deepen our understanding of emotional labor, but also to expand the methodological repertoire available to sport coaching or PE teaching settings.

## Data Availability

The original contributions presented in the study are included in the article/supplementary material, further inquiries can be directed to the corresponding author.
